# Deciphering the Proteotoxic Stress Responses Triggered by the Perturbed Thylakoid Proteostasis in Arabidopsis

**DOI:** 10.3390/plants10030519

**Published:** 2021-03-10

**Authors:** Kenji Nishimura, Reiko Nakagawa, Chisato Hachisuga, Yuri Nakajima Munekage

**Affiliations:** 1 Department of Bioscience, School of Science and Technology, Kwansei Gakuin University, Sanda 669-1337, Hyogo, Japan; chisato011219@gmail.com (C.H.); munekage@kwansei.ac.jp (Y.N.M.); 2 Laboratory for Phyloinformatics in RIKEN Center for Biosystems Dynamics Research (BDR), Kobe 650-0047, Hyogo, Japan; reiko.nakagawa@riken.jp

**Keywords:** chloroplast, heat stress, processive protease, proteostasis, stress granule, thylakoid membrane

## Abstract

Here, we explored heat dependent thylakoid FtsH protease substrates and investigated proteotoxicity induced by thermal damage and processive protease dysfunction on the thylakoid membrane. Through our thylakoid enriched proteome analysis and biochemical experiments, carbonylated stromal proteins were suggested as possible FtsH targets. Furthermore, we observed in the thylakoid fractions in the absence of FtsH stromal reactive oxygen species-detoxifying enzymes, as well as heat shock proteins and chaperones, which are known to be upregulated at the transcriptional level when this protease is absent, which is called the damaged protein response, resembling unfolded protein response in eukaryotic cells. Interestingly, the thylakoid-enriched high-density fractions included stromal translation factors and RNA-binding proteins, along with aminoacyl-tRNA synthetase, reminiscent of the formation of stress granules. Unexpectedly, extraplastid proteins such as mitochondrial chaperones, peroxidase, tricarboxylic acid cycle and respiratory chain enzymes, as well as cytosolic ribosomes, translation factors, heat shock proteins, antioxidants and metabolic enzymes, were also found deposited in the high-density fractions depending on the loss of thylakoid FtsH, with more prominent effects of thermal stress on the cytosolic proteins. This may reflect intracellular adaptation to the proteotoxic influences from the organelle.

## 1. Introduction

Several thousand proteins reside within the chloroplast, where they catalyze a wide range of metabolic reactions as well as biogenic processes, including photosynthesis and protein biogenesis [1]. Chloroplast proteins are mostly nucleus-encoded, and thus, synthesized by 80S ribosomes in the cytosol as preproteins having an N-terminal cleavable transit peptide facilitating their import into the organelle, while the remaining ~100 proteins are chloroplast-encoded and synthesized by 70S ribosomes within the plastid. These proteins are destined to the intraorganellar locations, i.e., stroma, envelopes and thylakoid membranes. 

The thylakoid membrane provides the site for photosynthesis, and their membrane structure is highly organized with photosynthetic protein machineries such as photosystems [2]. Photosynthetic complexes are often exposed to excessive light energy damaging their protein components, which must be removed and then replaced for optimal photosynthetic activities. Indeed, a protein quality control (PQC) mechanism repairing a primary target of photodamage, namely the reaction center D1 protein in the photosystem II (PSII), is present on the thylakoid membrane, called the PSII repair cycle [3]. 

Energy-dependent processive proteases contribute to chloroplast protein homeostasis (proteostasis) [4]. Filamentation temperature-sensitive H (FtsH) is an ATP-dependent processive protease of bacterial origin and functions on the thylakoid membrane as a zinc-dependent hetero-hexameric metalloprotease complex comprising four isoforms (FtsH1, FtsH2, FtsH5 and FtsH8), each consisting of an N-terminal transmembrane domain for membrane-anchoring, a AAA domain for ATP-driven substrate protein unfolding and a C-terminal proteolytic domain [5]. Loss of FtsH5/VAR1 or FtsH2/VAR2 but not FtsH1 or FtsH8 causes yellow leaf variegation (var) phenotypes [6]. Given the physiologic consequences of its absence, thylakoid membrane-anchored FtsH protease is considered pivotal in chloroplast proteostasis and plays a central role in PSII repair cycle [3]. Much has been learned about how FtsH functions in repairing the photodamaged PSII, while its additional role in proteostasis and stress response is not excluded. Indeed, leaf variegation cannot be explained solely by the FtsH function in the PSII repair cycle [7]. 

Chloroplasts are highly susceptible to heat stress and its major effects are inactivation of PSII, chlorophyll degradation, inhibition of Rubisco activity and failed chloroplast protein synthesis [8]. In particular, PSII is the most sensitive target for thermal damage among the photosynthetic machineries embedded on the thylakoid membrane, and heat stress causes destabilization of the oxygen-evolving complex in PSII affecting downstream photosynthetic electron transfer and ATP synthesis [9]. Given its location and accessibility to photosynthetic machineries on the thylakoids, the FtsH may well participate in possible PQC mechanisms maintaining thylakoid proteostasis during heat stress. 

Thus, we set out to test the possible mechanism through identifying putative thermal-dependent FtsH substrates by comparative thylakoid enriched proteome analysis of wild-type and *var2* mutant in response to heat exposure. In doing so we observed cellular proteome responses to the proteotoxicity provoked by heat stress and impaired processive proteolysis on the thylakoid membrane. 

## 2. Results

### 2.1. Comparing Thylakoid-Enriched Proteomes of var2 versus Wild-Type Plants in Response to Heat Exposure

**Figure 1:**
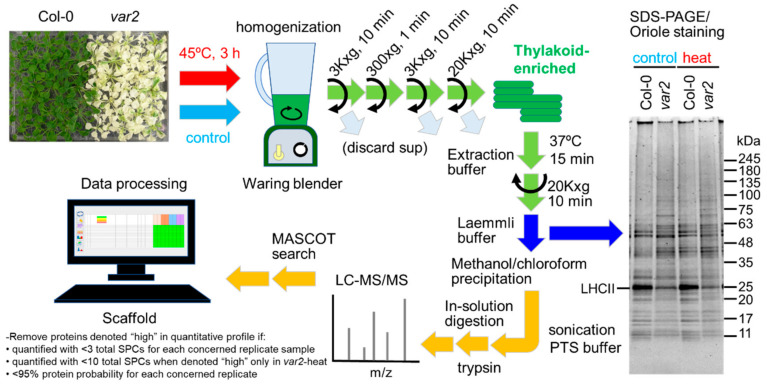
Workflow for analyzing thylakoid-enriched proteomes of *var2* mutant vs wild type Arabidopsis under normal and heat stress conditions. Wild-type Col-0 and *var2* mutant plants were grown on plates under normal conditions and then harvested before (control) or after heat stress (45 °C, 3 h), followed by homogenization. The homogenates were fractionated by a series of centrifugation processes to obtain thylakoid enriched fractions. Their proteins were extracted and analyzed by SDS-PAGE followed by Oriole staining (3 µg of proteins per lane). The protein extracts were methanol-chloroform precipitated for in-solution digestion with trypsin for LC-MS/MS analysis. Obtained mass spectra were subject to MASCOT search, and identified proteins were analyzed by Scaffold. Differentially accumulated proteins were searched using Scaffold with the indicated criteria (see method). SPC, spectrum count.

Aiming to explore thylakoid FtsH substrates under heat stress, we carried out a simple biochemical and proteomics experiments as follows (Figure 1). Wild-type and *var2-7* mutant [10] (hereafter *var2*) Arabidopsis plants grown for 4 weeks under normal growth conditions were either harvested immediately (non-treated) or exposed to heat stress at 45 °C for 3 h and then harvested (heat-treated), each followed by biochemical enrichment of thylakoid membrane fractions by simple low-speed centrifugation. Their proteins were extracted and separated on SDS-PAGE gel, showing specific differences in abundance between control and stress conditions for each genotype as well as between the two genotypes for both conditions (Figure 1). The protein extracts were then analyzed for identification and quantification using liquid chromatography-tandem mass spectrometry. Their absolute protein levels were estimated by exponentially modified protein abundance index (emPAI) [11] (Table S1). 

### 2.2. Verifying Thylakoid Proteostasis Impairment and Proteotoxic Stress Responses

**Figure 2:**
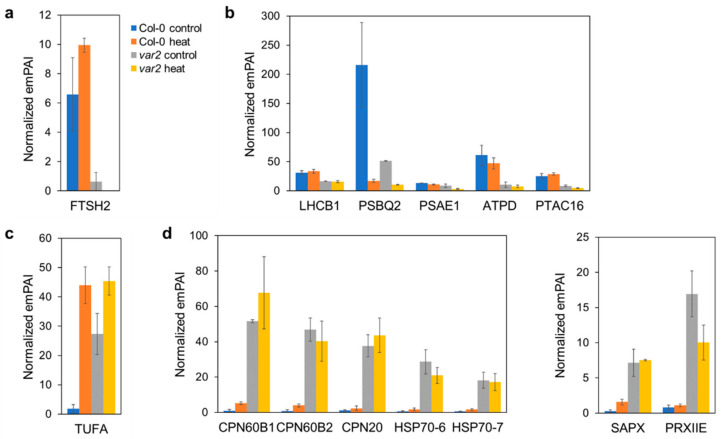
Verification of impaired thylakoid proteostasis and proteotoxic stress responses. Thylakoid-enriched fractions were prepared from heat-stressed and non-stressed wild-type and *var2* mutant plants, and their proteins were extracted and analyzed by nanoLC-MS/MS. Protein abundances were quantified and shown as emPAI-based normalized quantitative values (the mean of the two replicates +/− SD). For more details, refer to Table S1. (**a**) FtsH2 loss. Blue and orange bars indicate the wild-type plants before and after heat stress, respectively, and gray and yellow bars are the *var2* mutant before and after heat stress, respectively. (**b**) Misregulated thylakoid protein accumulation. PSBQ2, a subunit of heat-labile oxygen-evolving complex in the thylakoid membrane, was decreased by heat stress and loss of FtsH. Decreased accumulation was also observed for photosynthesis-related and nucleoid-associated proteins. (**c**) An aggregate-prone stromal protein TUFA as a proxy for proteotoxic stresses. (**d**) Recruitment of stromal chaperones and ROS-detoxifying enzymes to the thylakoid membrane in response to the FtsH loss. Left panel indicates chaperone protein abundances. Right panel shows the abundances of ROS-detoxifying enzymes.

As expected, we confirmed the FtsH2 deficiency in the *var2* mutant thylakoid fractions (Figure 2a). Levels of thylakoid proteins such as a light harvesting complex II proteins (LHCB1 and LHCB5), PSI/PSII components (PSBQ2, PSAE1 and PSAE2) and ATP synthase subunits (ATPD, atpB and atpF) were affected in the *var2* mutant (Figure 2b and Figure S1a). PSBQ2 decrease was detected even in the wild type plants under heat stress, consistent with the oxygen-evolving complex as susceptible to heat damage [9]. Abundance of a plastid transcriptionally active chromosome component PTAC16 was also reduced in the *var2* thylakoid. PTAC16 is a nucleoid protein, but its function is unknown [12,13].

Importantly a stromal protein prone to aggregation, TUFA/Rabe1b, was found to over-accumulate in the thylakoid fractions from the wild type plants under heat stress (Figure 2c). Rabe1b is the translation elongation factor Tu (EF-Tu) in chloroplasts. EF-Tu is a conserved GTP-binding protein that facilitates the entry of aminoacyl-tRNAs into the ribosome during mRNA decoding [14], while it can also function as a molecular chaperone preventing thermal protein aggregation [15]. Rabe1 is involved in heat tolerance but is heat labile and prone to heat-induced aggregation, which likely inactivates its elongation factor function [16]. Rabe1b aggregates are detected in insoluble fractions separated by low-speed centrifugation (2200× *g*, 5 min) of leaf cell extracts from heat stressed plants. Slow centrifugation also effectively enriches high-density thylakoid membrane fractions; we obtained thylakoid preparations by spinning down at a comparable speed (3000× *g*, 10 min), and thus, the aggregates could co-sediment with the thylakoid membranes in our experiments. 

Rabe1b aggregation was also observed in the thylakoid fractions of the *var2* mutant plant under normal as well as heat stress conditions (Figure 2c), indicating proteotoxic stresses occurring in these mutant background and environmental condition. Indeed, stromal chaperones (CPN60B1, CPN60B2 and CPN20) and heat shock proteins (HSP70-6 and HSP70-7) involved in protein folding were found to be located to these thylakoid fractions (Figure 2d). Similar relocation has been reported for Clp, another major processive protease in chloroplasts; this protease complex is normally stroma-localized, but is recruited to the thylakoid membrane when the thylakoid FtsH function is disrupted [17]. In addition, pyruvate dehydrogenase (PDH) complex E2 subunit (LTA2) was also thylakoid-associated in the *var2* mutant (Figure S1b). The PDH multienzyme complex is present in stroma where it catalyzes sequential reactions, converting pyruvate into acetyl coenzyme A and NADH for fatty acid biosynthesis [18,19]. 

Stromal ROS-scavenging enzymes were also detected in the *var2* thylakoid fractions (Figure 2d). Excess ROS accumulation can cause detrimental toxic effects, including oxidative damage to nucleic acids, lipids and proteins in chloroplasts, and thus, are regulated by enzymatic as well as nonenzymatic mechanisms [20]. The stromal ascorbate peroxidase (SAPX) is a heme-binding peroxidase that oxidizes ascorbate to reduce hydrogen peroxide to water, while the type II peroxiredoxin (PRXIIE) is a heme-independent thiol peroxidase in stroma that requires a cysteinyl thiolate for the reduction of peroxide compounds [21]. Their thylakoid relocation can be explained by the observed ROS accumulation in *var2* chloroplasts due to impaired photosystem quality control (Kato et al., 2009). Thylakoid association of a stromal iron-storage protein ferritin (FER1), which is considered an antioxidant [22], may suggest another ROS-scavenging mechanism in the *var2* mutant (Figure S1b).

Remarkably, in the *var2* mutant, the thylakoid recruitment of organellar oligopeptidase (OOP) and two ROS-detoxifying enzymes, glutathione S-transferase F8 (GSTF8) and glutamate-cysteine ligase (GSH1), was further enhanced during heat stress (Figure S1b), likely reflecting heat-responsive FtsH-dependent proteotoxicity. OOP cleaves proteolytic fragments as well as transit peptides ranging from 8 to 23 amino acids in length, acting downstream of PreP and processive proteases [23,24]. GSTF8 catalyzes the conjugation of reduced glutathione to hydroperoxides including oxylipins for detoxification [25]. GSTF8 expression is ROS-inducible and the protein level is elevated in the *var2* chloroplasts [26]. GSH1 is the rate-limiting enzyme in glutathione synthesis [27]. 

Taken together, our proteomic observations suggested impaired thylakoid proteostasis and proteotoxic stress responses. 

### 2.3. Oxidative Stress-Dependent Protein Accumulation

**Figure 3:**
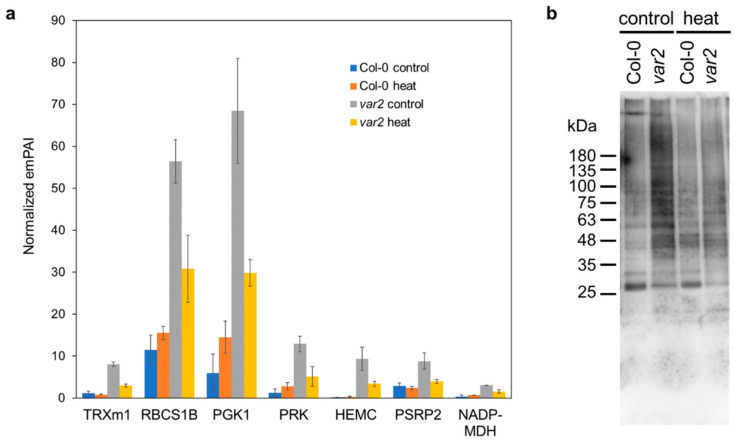
Oxidative stress-dependent protein accumulation in *var2.* (**a**) Stromal proteins affected by the FtsH defect. Protein abundances were shown as emPAI-based normalized quantitative values (the mean of the two replicates +/− SD). For details, refer to Table S1. (**b**) Immunoblot analysis showing carbonylated proteins accumulating in the *var2* mutant under normal conditions rather than stress conditions. The carbonylated proteins were chemically modified with 2,4-dinitrophenylhydrazine (DNPH) and detected using anti-DNP antibody. Each lane contains 3 µg of protein extracts from the thylakoid fractions.

A set of stromal proteins were over-accumulated in the *var2* thylakoid fractions even under normal conditions, but the heat stress treatment attenuated their thylakoid accumulation (Figure 3a). These included m-type thioredoxin (TRX m1), Calvin cycle enzymes (RBCS1B, PRK and PGK1), tetrapyrrole biosynthesis enzyme (HEMC), ribosome subunit (PSRP2) and NADPH-dependent malate dehydrogenase (NADP-MDH). Chloroplast TRXs are protein oxidoreductases that catalyze the reduction of disulfide bonds in target proteins to modulate and restore their structure and activity, forming a complex regulatory network involved in redox homeostasis for controlling diverse metabolic pathways, chloroplast biogenesis and oxidative stress responses [28,29,30]. 

The m-type TRXs (TRX m) are the most abundant isoforms and regulate the Calvin-Benson cycle, tetrapyrrole biosynthesis and protein biogenesis [31,32,33]. NADP-MDH is a malate valve enzyme involved in the export of excess reducing power from chloroplasts through the envelope-localized dicarboxylate transporter to the cytosol [34] and is also activated by TRX m [35]. Importantly, TRX m can reactivate denatured glucose-6 phosphate dehydrogenase and even prevent heat-induced MDH aggregation [36]. Given its chaperone-like activities, TRX m1 may be recruited to oxidatively-damaged and unfolded stromal proteins, which otherwise could be degraded by FtsH, deposited in the thylakoid fractions, where it could help resolve the aggregates. Our immunoblot analysis, indeed, showed overaccumulation of oxidized proteins in the thylakoid fraction from the *var2* mutant compared to the wild-type plants (Figure 3b). In this experiment, we used a chemical, namely, 2,4-dinitrophenylhydrazine (DNPH), to specifically probe ROS-induced carbonylated proteins and detected the chemically modified proteins with anti-DNP antibody. Interestingly, the protein carbonylation was much lower in the heat-treated rather than in the non-treated *var2* mutant, as observed for the accumulation of the above-mentioned stromal proteins. 

### 2.4. Altered Stromal Protein Distribution

**Figure 4:**
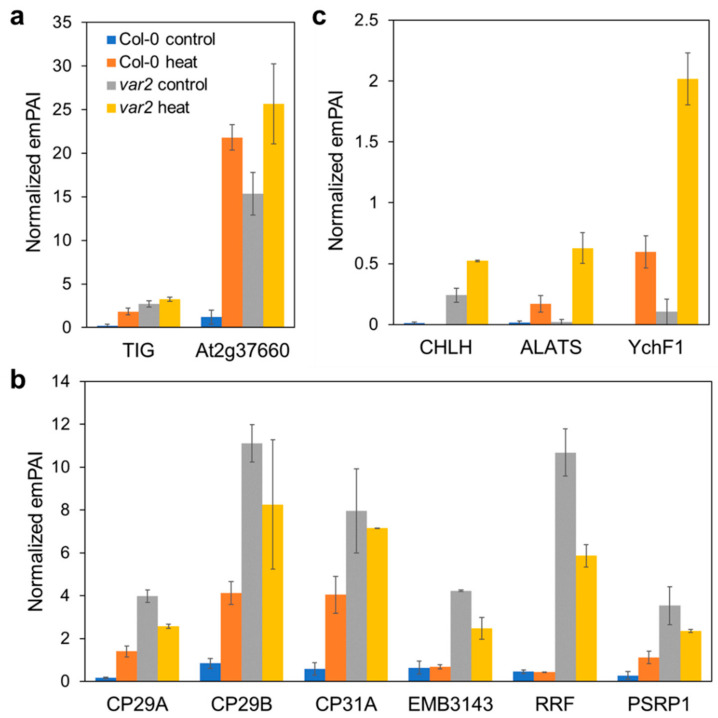
Altered stromal protein homeostasis in *var2.* The data represent emPAI-based normalized quantitative values (the mean of the two replicates +/− SD). For detailed information, see Table S1. (**a**) Other aggregate-prone stroma proteins. TIG is chloroplast trigger factor. At2g37660 is an unknown protein. (**b**) Stromal proteins affected more by the FtsH loss than by heat stress alone. CP29A/B and CP31 are RNA-binding proteins. EMB3143 is also known as YCF54 or LCAA (Low Chlorophyll Accumulation A) and functions as a scaffold protein for a tetrapyrrole biosynthesis enzyme, magnesium protoporphyrin monomethylester cyclase complex. RRF is chloroplast ribosome recycling factor. PSRP1 (Plastid specific ribosomal protein) is not a ribosome subunit but a ribosome binding factor. (**c**) Heat-enhanced FtsH effects on the stromal protein abundances. CHLH is H subunit of magnesium chelatase involved in chlorophyll biosynthesis and is also known as GUN5 (genome uncoupled 5). ALATS is alanyl-tRNA synthetase. YchF1 is a GTPase.

Another set of stromal proteins were also affected by thermal stress, loss-of-FtsH and the combined proteotoxicity. A chloroplast trigger factor TIG over-accumulated in the thylakoid fractions from *var2* mutant plants before and after heat stress as well as the wild type plants exposed to heat stress (Figure 4a). TIG is involved in the correct maturation of nascent polypeptides during protein biogenesis through its specialized chaperone function, which is related to its limited association with translating ribosomes compared to its prokaryotic counterpart [37]. At2G37660 was also detected in the thylakoid fractions from the stressed wild-type as well as the *var2* mutant plants under both conditions, but its function is uncharacterized (Figure 4a). 

Similarly to CPN/HSPs and SAPX/PRXIIE, some stromal proteins were highly accumulated in the thylakoid fractions in the *var2* mutants, as exemplified by ribonucleoproteins (CP29A, CP29B and CP31A), scaffold protein EMB3143/YCF54/LCAA, ribosome binding factor PSRP1 (AT5G24490) and ribosome recycling factor (RRF) (Figure 4b). CP29B function has yet to be determined, while CP29A and CP31A bind to large sets of chloroplast transcripts to ensure their stability and facilitate specific posttranscriptional processes under low temperatures [38]. Even without cold stress, CP31A loss alone is known to influence editing efficiency and stability of specific chloroplast mRNAs including the ndhF transcript [39]. NDH subunits accumulation seemed to be compromised in the *var2* mutant thylakoids (Table S1), suggestive of inactive CP31A. PSRP1 is not a ribosomal protein, but binds within the subunit interface of the 70S ribosome where the peptidyl transferase center is located [40]. This binding induces conformational changes within ribosome components to stabilize the 70S structure in a dormant state during stresses, which can be competitively dissolved by RRF together with another elongation factor, EF-G. The presence of PSRP1 and RRF in the thylakoid may mirror compromised protein synthesis in proteotoxic conditions. These stromal proteins seem not to be upregulated in the *var2* mutant chloroplasts [26], suggesting that their increased thylakoid association is independent of the UPR-like response. 

Mg-chelatase subunit H (CHLH/GUN5) was also found associated to the thylakoid fractions in the *var2* mutants, but its association was more pronounced under heat stress, unlike the above ribonucleases and translation factors (Figure 4c). Interestingly, alanyl-tRNA synthetase (ALATS) as well as YchF1/ENGD GTPase showed heat-dependent relocation to the thylakoid fractions, and this was more prominent in the *var2* than in the wild-type background, even though the FtsH defect alone seemed not to affect the ALATS/YchF1 localization (Figure 4c).

### 2.5. Consequences on Extra-Plastid Proteostasis

**Figure 5:**
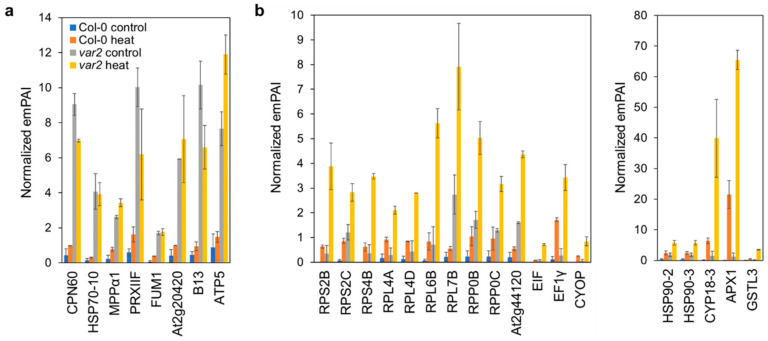
Consequences on mitochondria and cytosolic proteostasis. All the bar graphs indicate normalized emPAI-based quantitative values (the mean of the two replicates +/− SD). For further information, see Table S1. (**a**) Mitochondrial proteins affected by proteotoxic stresses occurring on the thylakoids. The affected proteins were chaperones (CPN60, HSP70-10), peptidase (MPPα1), peroxiredoxin (PRXIIF), fumarate hydratase (FUM1), succinate-CoA ligase subunit (At2g20420), NADH dehydrogenase (Complex I) subunit (B13) and ATP synthase (Complex V) subunit (ATP5) in mitochondria. (**b**) Cytosolic proteins deposited in response to the proteotoxicity invoked by heat and FtsH loss. Left panel shows 80S ribosome subunit proteins (RPS2B/2C/4B, RPL4A/4D/6B/7B/RPP0B/0C and At2g44120), translation factors (EIF and EF1γ) and oligopeptidase (CYOP). Proteins related to protein folding (HSP90-2/3 and CYP18-3) and ROS-detoxification (APX1 and GSTL3) are in the right panel.

Non-plastid proteins were also detected in our thylakoid preparations (Table S1); the heat stress could influence the stability of proteins residing outside the chloroplast, and thus, their aggregates might be contaminated in these high-density membrane fractions. Nevertheless, a set of the extra-plastid proteins were enriched in the thylakoid fractions in a manner depending on the FtsH absence before and after heat stress (Table S1). These proteins were mostly cytosolic and to a lesser extent mitochondrial. Mitochondrial chaperones, processing peptidase, type II peroxiredoxin, TCA cycle enzymes (fumarate hydratase and succinate-CoA ligase), respiratory chain complexes I and V subunits were co-sedimented with thylakoid fractions from the *var2* mutant both with or without heat exposure (Figure 5a). Interestingly, cytosolic 80S ribosome, chaperones, oligopeptidase, ROS-scavengers (APX1 and GSTL3), cysteine synthase, lectin, glycolytic enzymes (fructose bisphosphate aldolases, phosphoglucomutase) and glyoxylate-dicarboxylate metabolism enzyme (oxalate-CoA ligase) were more prominent in the heat-exposed *var2*′s thylakoids fractions (Figure 5b and Figure S1c). We note that the latter fractions also included translation initiation factor G (EIF) and poly(A)-binding protein (PAB8) (Figure 5b, Table S1), the factors defining heat stress granules (HSGs) [41]. HSGs are protein/poly(A)^+^-RNA structures that form upon heat exposure, arresting translation and storing mRNAs to limit the entry of newly-synthesized proteins into overloaded proteostasis systems [41,42]. 

## 3. Discussion

A conserved FtsH processive protease plays a central role in thylakoid proteostasis; thus, loss of its function could cause proteotoxic stress. Heat stress also disturbs proteostasis and primarily injures the photosynthetic machinery on the thylakoid membrane in plants. In our proteomics study aiming to identify heat-dependent FtsH clients (Figure 1), we observed the proteome responses to proteotoxicity imposed by thermal stress and failed processive proteolysis on the thylakoid membrane. Employing TUFA/Rabe1b, a superaggregating protein in stroma, as a proxy for the proteotoxicity (Figure 2c), we found many stromal proteins deposited in the thylakoid-enriched fractions in response to the stresses (i.e., Heat exposure, FtsH deficiency and the combined effects) (Figure 2, Figure 3 and Figure 4 and Figure S1a,b). Whether these deposited stromal proteins or aggregates are physically bound to the thylakoid membrane is unclear; however, their relocation, depending on the FtsH defects, suggests either that the deposited stromal proteins are normally removed by the action of the FtsH protease on the thylakoid membrane or that the FtsH defects on the thylakoid cause the stromal protein aggregation (e.g., via concomitant ROS-induced damage). Considering that the membrane surface can accelerate and/or stabilize protein aggregation [43] and a large surface area is a hallmark feature of the thylakoid membrane [44], stromal protein aggregates may be inclined to occur and grow on the thylakoid surface. Their thylakoid localization merits further examination.

Disrupted organellar proteostasis activates retrograde signal transduction to induce expression of nuclear genes encoding PQC-related proteins including chaperones, heat shock proteins and proteases, which is known as unfolded protein response (UPR) [45,46]. Chloroplasts UPR was first discovered in the model green alga Chlamydomonas where the conditional loss of the Clp protease triggers expression of nuclear-encoded autophagy- and PQC-related genes [47]. Loss of the FtsH function causes a UPR-like response inducing the expression of chaperones, proteases and ROS-detoxifiers for PQC [26]. We also detected these PQC proteins in the *var2* mutant thylakoid fractions (Figure 2 and Figure 4), many of which were likely recruited from stroma to the thylakoid membrane, where the proteotoxic challenges were posed. PDH’s E2 subunit LTA2 was also relocated to the mutant thylakoid fractions (Figure S1b). Interestingly in green algae E2 subunit has an alternate function as an RNA binding protein involved in membrane targeting and translation of the psbA mRNA for PSII reaction center D1 protein [48]. This moonlighting function of E2 might well be conserved in higher plant LTA2; this would be consistent with thylakoid FtsH function in replacing damaged D1 in the PSII repair cycle. 

Importantly, stromal ribonucleoproteins, namely CP29A, CP29B and CP31A, were found in the mutant thylakoid fractions (Figure 4b). RNA-binding proteins are known to form heat-triggered aggregates resembling HSGs [41]. Importantly, CP29A is detected in recently-identified heat-induced chloroplast stress granules, where it is suggested to function as a scaffold protein for their assembly [49]. Given its presumable inactive state, CP31A, and perhaps, CP29B may form similar stress-dependent granules or protein aggregate centers (PACs), which could be facilitated by chaperones including HSP70 family proteins as observed in yeast [41,50]. The chaperone-assisted PAC assembly serves as the nucleation core for aggregation or stress granule formation to prevent proteolysis and helps resume growth after stress [50]. Likewise, aminoacyl-tRNA synthetases is prone to heat-triggered aggregation albeit without losing their activity and fidelity [41], supporting the idea that ALATS could be deposited as such stress-driven aggregates in the thylakoid fractions (Figure 4c). We found an additional translation factor RRF along with TUFA/Rabe1b deposited in the thylakoid fractions from the *var2* mutant (Figure 4a). Heat stress-dependent accumulation of translation elongation and termination factors serves as a hallmark for stress granules formation [51]; thus, similar stress granules likely form in chloroplasts when thylakoid FtsH is absent. The stress granule formation deserves to be scrutinized. Stress granules also employ signaling proteins, including scaffold proteins, phosphatase, ribonucleoproteins, methyltransferase and GTPase, thereby functioning as signaling hubs [52]. Rabe1b loss compromises heat stress signaling and suppresses *var2* leaf variegation phenotype [53]. 

Metabolic enzymes, such as RBCS, PGK1, PRK, HEMC and NADP-MDH, along with a ribosome subunit PSRP2, all of which are normally stroma-localized, were highly accumulated in the thylakoids of the *var2* mutants before rather than after heat stress (Figure 3a). Their accumulation was correlated with the carbonylated protein abundances (Figure 3b), likely suggesting these enzymes and ribosome being oxidatively damaged. This was supported by the co-localization of a stromal antioxidant chaperone, namely TRX m1, in the membrane fractions (Figure 3a). Recently, oxidative posttranslational protein modification has been suggested to trigger FtsH-dependent proteolysis [54]. We, thus, propose that some if not all the above stromal enzyme and subunit proteins may be potential FtsH substrates. Considering its stroma-facing chaperone-protease domain [6], FtsH could recognize and destroy stromal proteins when they appear close to or on the thylakoid membrane. Mechanistic underpinning of its substrate recognition and degradation warrants further investigation. We note that we could not find overaccumulation of thylakoid resident proteins including photosystem subunits in the *var2* mutants either under normal or heat stress conditions. Given its upregulation and recruitment to the thylakoid membrane in *var2* mutants [17,26], the Clp protease might also contribute to thylakoid protein quality control in a compensatory manner.

We did detect cytosolic and mitochondrial proteins in the thylakoid-enriched membrane fractions depending on the lack of FtsH (Figure 5). These included metabolic enzymes as well as chaperones, peptidases and ROS-quenchers, perhaps reflecting FtsH-loss-triggered proteotoxic stresses prevailing outside the chloroplast, and thus, the cell’s adaptation strategy to the intraorganellar proteotoxic influences. Moreover, the observation that cytosolic translation factors and RNA-binding proteins along with ribosomal proteins were heat-aggregated depending on the FtsH loss may reflect the formation of HSGs, attenuating translation in cytosol. Since most chloroplast proteins are cytosolically synthesized, this translation attenuation would help alleviate accumulation of damaged proteins in chloroplasts. Indeed, we found protein carbonylation was much less in the *var2* mutant after than before heat stress (Figure 3b). This may be related to the fact that loss of cytosolic ribosomal proteins, albeit to varying degree, enhances *var2* leaf variegation [55]. Otherwise, some but not PQC-related extra-plastid proteins might be supposed to be degraded inside the chloroplast when damaged or aggregated. Interestingly, in yeast cytosolic aggregate-prone proteins engage with mitochondrial import machinery on the outer membrane and enter mitochondrial intermembrane space and matrix for degradation, indicating the presence of mitochondria-mediated cytosolic proteostasis mechanism [56]. Whether and how the cytosolic proteins found in the thylakoid fractions of the heat-treated plants lacking FtsH could be relocated to the thylakoid membranes remain unclear, but a possible involvement of FtsH in their clearance may deserve future evaluation. 

## 4. Materials and Methods

### 4.1. Plant Materials, Growth Condition and Stress Experiment

The wild-type *Arabidopsis thaliana* Columbia-0 ecotype plants was used as a control. The *var2* mutant seeds were kindly provided by Dr. Wataru Sakamoto (Institute of Plant Science and Resources, Okayama University). Plants were grown for 4 weeks on plates containing Murashige and Skoog plant salt mixture (Wako), pH 5.7, 8 µM nicotinic acid, 5 µM pyridoxine hydrochloride, 30 µM thiamine hydrochloride, 555 µM myoinositol, 3% sucrose and 0.25% Phytagel (Sigma-Aldrich) at 24 °C under 100 µmol m^−2^ s^−1^ of white LED light for 12/12-h photoperiod cycles. For heat stress, the plates were placed in an incubator at 45 °C in the dark for 3 h. 

### 4.2. Thylakoid Isolation and Protein Extraction

Before or after heat stress treatment, plants were harvested and ground in ice-cold homogenization buffer (50 mM hepes-KOH, pH 7.0, 5 mM MgCl_2_, 10 mM NaCl, 2 mM EDTA) with Waring blender, and the homogenates were passed through Miracloth and centrifuged at 3000× *g* for 10 min at 4 °C to pellet crude thylakoid fractions. The crude fractions were resuspended with the homogenization buffer and then centrifuged at 300× *g* for 1 min at 4 °C, and the supernatant was further centrifuged at 3000× *g* for 10 min at 4 °C. The resulting pellets were resuspended with ~1 mL of the homogenization buffer and centrifuged at 20,000× *g* for 10 min at 4 °C to obtain the thylakoid-enriched fractions. 

These fractions were resuspended with extraction buffer containing 50 mM Tris-HCl, pH8, 1% SDS and 1% Protease inhibitor cocktail for plant cell and tissue extracts (Sigma-Aldrich) and incubated at 37 °C for 15 min, followed by centrifugation at 15,000× *g* for 10 min at room temperature. The resulting supernatant was collected and combined with 4 × Laemmli buffer (Bio-Rad) for SDS-PAGE and immunoblotting analyses. For LC-MS/MS analysis, these protein solutions were precipitated with methanol and chloroform. 

### 4.3. SDS-PAGE and Immunoblotting

Proteins were separated on the e-PAGEL precast gels (10 to 20% acrylamide gradient; ATTO) and visualized with Oriole fluorescent gel stain (Bio-Rad) and ultraviolet exposure. Carbonylated proteins were detected by immunoblotting with protein carbonyls western blot detection kit (Cosmo Bio Co., Ltd., Tokyo, Japan) according to the manufacturer’s instructions.

### 4.4. LC-MS/MS Analysis and Data Processing

The protein pellets were dissolved in PTS buffer (12 mM Sodium deoxycholate, 12 mM sodium lauryl sulfate, 25 mM ammonium bicarbonate buffer) with brief sonication, and then digestion with 10 μg/mL modified trypsin (MS grade, Thermofisher Scientific) was performed at 37 °C for 16 h. The digested peptide solutions were subjected to reduction with 10 mM DTT at 56 °C for an hour, and alkylation with 55 mM iodoacetamide at room temperature for 45 min. After desalting the peptide solutions with an in-house made C18 Stage-tip, it was dried under a vacuum, and dissolved in 2% acetonitrile and 0.1% formic acid. The concentration of the peptide solution was quantified by the Qubit Protein Assay Kit (Themofisher scientific, Waltham, MA, USA).

The 100 ng peptides mixtures were then fractionated by C18 reverse-phase chromatography (3 μm, ID 0.075 mm × 250 mm, Acclaim PepMap 100 C18 column; Thermofisher Scientific, ADVANCE UHPLC; AMR Inc., Tokyo, Japan) and measured by a hybrid linear ion trap mass spectrometer (LTQ Orbitrap Velos Pro; Thermo Fisher Scientific, Waltham, MA, USA) with Advanced Captive Spray SOURCE (AMR Inc., Tokyo, Japan). The peptides were eluted at a flow rate of 200 nL/min with a linear gradient of 5–35% solvent B over 155 min, then to 90% for 15 min and finally held at 5% for the last 10 min. The composition of Solvent A and B were 0.1% TFA in water and 100% acetonitrile, respectively. The mass spectrometer was programmed to carry out 13 successive scans consisting of, first, a full-scan MS over the range 350–1800 m/z by FT-ICR at a resolution of 60,000, and second to thirteenth automatic data-dependent MS/MS scans of the top 12 abundant ions obtained in the first scan by ion trap. MS/MS spectra were obtained by setting the relative collision energy of 35% CID and exclusion time of 20 sec for molecules of the same m/z value range. The molecular masses of the resulting peptides were searched against the *Arabidopsis thaliana* amino acid sequence dataset (Uniprot Proteome *Arabidopsis thaliana* 2020.05.04 downloaded; Proteome ID UP000006548, 39,351 sequences; 16,654,261 residues) using the MASCOT version 2.6 (Matrix Science) with a false discovery rate (FDR) set at 0.01. Carbamidomethylation of cysteine was set as a fixed modification, oxidation of methionine, and acetylation of protein N-termini were included as variable modifications. The number of missed cleavages site was set as 2. The obtained results were visualized using Scaffold 4.8.9 (Proteome Software). A minimum threshold for peptide (95%) and protein (99%) in addition to the identification of a minimum of three unique peptides were considered as hits after normalization with untagged control spectra. 

Highly accumulated proteins were considered based on the quantitative profile in Scaffold. To improve the robustness, the following proteins were omitted: (1) proteins that were denoted “high” but quantified with less than three total spectrum counts for each concerned replicate, (2) proteins that were denoted “high” only in the heat-treated *var2* mutants but quantified with less than 10 total spectrum counts and (3) proteins that were denoted “high” with less than 95% protein probability for each concerned replicate. Information on curated protein locations in the cell were retrieved from the PPDB ((http://ppdb.tc.cornell.edu/) accessed on 9 March 2021).

## Data Availability

The raw mass spectrometry data have been deposited in the jPOST repository with the dataset identifiers PXD022899 for ProteomeXchange and JPST001024 for jPOST (https://repository.jpostdb.org/preview/15881222075fc9a1b73abce (accessed on 30 December 2020); Access key: 3414).

## Supplementary Materials

The following are available online at https://www.mdpi.com/2223-7747/10/3/519/s1, Table S1. Differentially accumulated proteins in response to the proteotoxic stresses. Figure S1. Additional sets of proteins that were differentially accumulated in response to the proteotoxic stresses, related to Figure 2 and Figure 5. Each bar graph shows protein abundances shown as normalized emPAI-based quantitative values (the average of the two replicates +/− SD). More details can be found in Table S1. a. Accumulation of additional photosynthesis-related proteins. atpB and atpF are plastid-encoded, while the others are nucleus-encoded. Accumulation of a subset of thylakoid proteins was heat responsive in the wild-type but not in the var2 (right panel). b. Recruitment of other stromal proteins to the thylakoid membrane in response to proteotoxic stresses. Left panel indicates proteins related to iron storage (FER1) and RNA binding (LTA2). Their accumulation in the thylakoid fractions depends on the FtsH loss. Right panel displays oligopeptidase (OOP) and another set of proteins involved in ROS detoxification (GSH1 and GSTF8), whose accumulation in the thylakoid fractions is heat responsive but further enhanced by the lack of FtsH. c. Other cytosolic proteins cosedimented in the thylakoid enriched fractions from heat-stressed var2 mutants. These includes cysteine synthase (OASA1), Oxalate-CoA ligase (AAE3), fructose bisphosphate aldolases (FBA6/8), phosphoglucomutase (PGM), copper transport protein (CCH), bifunctional enolase 2 (ENO2), nucleosome assembly factor (NAP1) and lectin protein (PBP1).
