# Supplementary material for: Deciphering the Proteotoxic Stress Responses Triggered by the Perturbed Thylakoid Proteostasis in Arabidopsis
Source: Plants (Basel). 2021 Mar 10;10(3):519. doi: 10.3390/plants10030519 (PMC8001255; doi:10.3390/plants10030519)
Supplement: Supplementary file 1 [file plants-10-00519-s001.zip › FigureS1-final.docx]

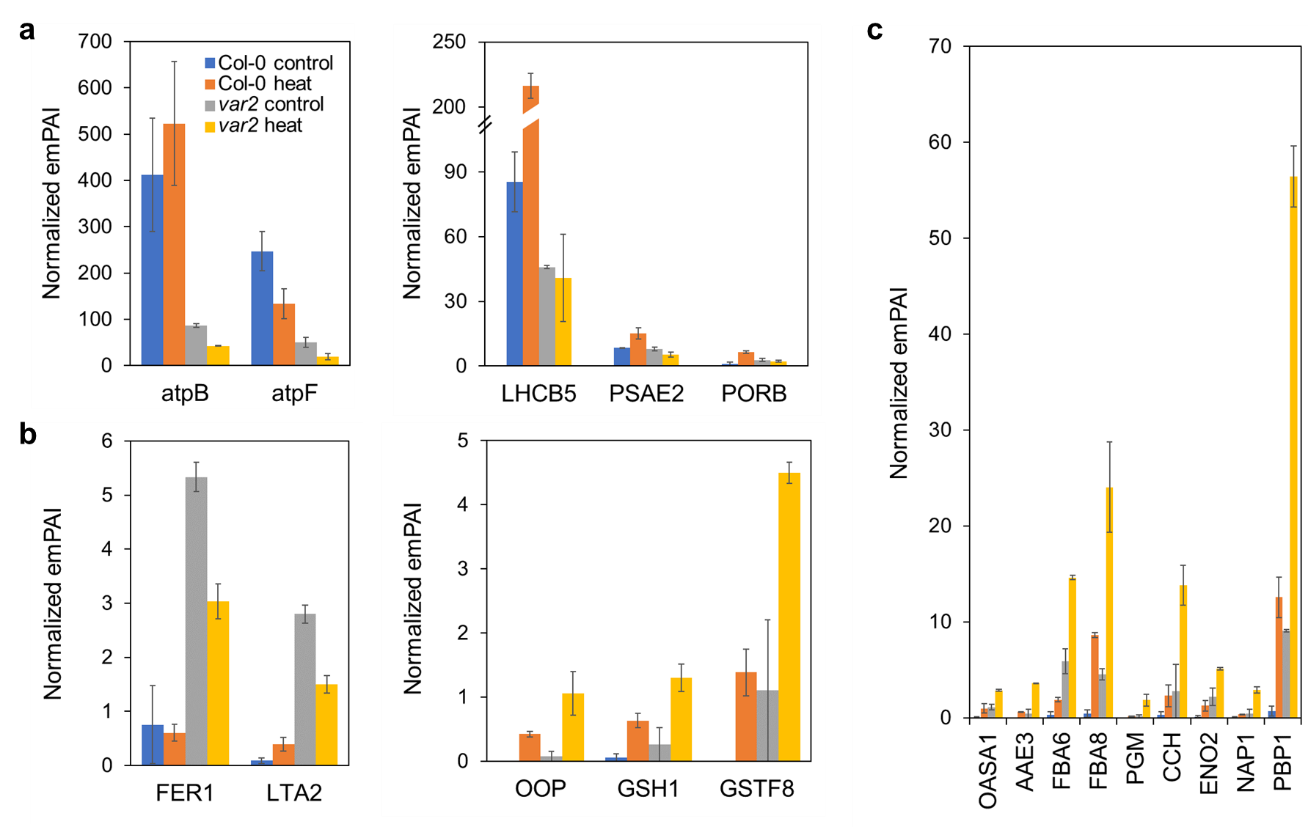


**Figure S1.** Additional sets of proteins that were differentially accumulated in response to the proteotoxic stresses, related to Figures 2 and 5.

Each bar graph shows protein abundances shown as normalized emPAI-based quantitative values (the average of the two replicates +/- SD). More details can be found in Table S1.

a. Accumulation of additional photosynthesis-related proteins. atpB and atpF are plastid encoded, while the others are nucleus encoded. Accumulation of a subset of thylakoid proteins was heat responsive in the wild-type but not in the *var2* (right panel).

b. Recruitment of other stromal proteins to the thylakoid membrane in response to proteotoxic stresses. Left panel indicates proteins related to iron storage (FER1) and RNA binding (LTA2). Their accumulation in the thylakoid fractions depends on the FtsH loss. Right panel displays oligopeptidase (OOP) and another set of proteins involved in ROS detoxification (GSH1 and GSTF8), whose accumulation in the thylakoid fractions is heat responsive but further enhanced by the lack of FtsH.

c. Other cytosolic proteins cosedimented in the thylakoid enriched fractions from heat-stressed var2 mutants. These includes cysteine synthase (OASA1), Oxalate-CoA ligase (AAE3), fructose bisphosphate aldolases (FBA6/8), phosphoglucomutase (PGM), copper transport protein (CCH), bifunctional enolase 2 (ENO2), nucleosome assembly factor (NAP1) and lectin protein (PBP1).
